# Navigating Uncertainty: The Role of Mood and Confidence in Decision-Making Flexibility and Performance

**DOI:** 10.3390/bs14121144

**Published:** 2024-11-28

**Authors:** Claudio Lavín, Roberto García, Miguel Fuentes

**Affiliations:** 1 Departamento de Psicología, Universidad Autónoma de Chile, Región Metropolitana, Santiago 7500912, Chile; 2 Facultad de Psicología, Universidad Diego Portales, Región Metropolitana, Santiago 8320000, Chile; 3 Santa Fe Institute, Santa Fe, NM 87501, USA; 4 Instituto de Investigaciones Filosóficas—SADAF, Buenos Aires 1188, Argentina; 5 Instituto de Sistemas Complejos de Valparaíso, Artillería 470, Cerro Artillería, Valparaíso 2340000, Chile

**Keywords:** decision-making, prediction-error, uncertainty, confidence, mood

## Abstract

Dealing with uncertainty is a pivotal skill for adaptive decision-making across various real-life contexts. Cognitive models suggest that individuals continuously update their knowledge based on past choices and outcomes. Traditionally, uncertainty has been linked to negative states such as fear and anxiety. Recent evidence, however, highlights that uncertainty can also evoke positive emotions, such as surprise, interest, excitement, and enthusiasm, depending on one’s task expectations. Despite this, the interplay between mood, confidence, and learning remains underexplored. Some studies indicate that self-reported mood does not always align with confidence, as these constructs evolve on different timescales. We propose that mood influences confidence, thereby enhancing decision flexibility—defined as the ability to switch effectively between exploration and exploitation. This increased flexibility is expected to improve task performance by increasing accuracy. Our findings support this hypothesis, revealing that confidence modulates exploration/exploitation strategies and learning rates, while mood affects reward perception and confidence levels. These findings indicate that metacognition entails a dynamic balance between exploration and exploitation, integrating mood states with high-level cognitive processes.

## 1. Introduction

Navigating uncertainty is crucial for adaptive decision-making across various real-life contexts. Uncertainty is inherently challenging because it involves a lack of clear information or ambiguous signals about future events’ probabilities [1], requiring strategies to be tested against unknown circumstances. Various cognitive models suggest that individuals gradually update their knowledge about their environment based on the history of choices and outcomes [2]. Sensitivity to environmental uncertainty is essential for appropriately weighting past and recent experiences, guiding both the exploration of new strategies and the exploitation of optimal options [3]. In real-life situations, such as managing economic volatility, an individual’s approach to uncertainty can shape the strategies of large-scale organizations, including countries (in the case of governments) or companies (in the case of managers) [1].

The exploration–exploitation dilemma has been used as an experimental framework to understand how people handle uncertainty, leveraging high-level cognitive functions such as metacognition and prospection [4]. Evidence indicates a connection between long-term thinking (operationalized as temporal discounting) and directed exploration, highlighting the long-term benefits of this strategy compared to inflexible decision-making [5]. In cognitive neuroscience, substantial evidence links long-term thinking with prefrontal cortex activity associated with executive processes [6,7]. Specifically, studies using neuroimaging methods suggest that the dorsal anterior cingulate cortex (dACC) monitors decision uncertainty. In contrast, the lateral frontopolar cortex (lFPC) is involved in the metacognitive control of decision adjustments, supporting the notion of a prefrontal metacognitive monitoring process independent of the decision process itself [6,7]. In practical scenarios, such as managers dealing with economic volatility, cognitive control over impulsive behavior is traditionally linked to adaptive behavior [8]. However, recent evidence suggests that impulsivity can be beneficial depending on the available choices, task structure, and volatility [8]. In management, for instance, the negative impact of uncertainty can be mitigated by managers’ tolerance for ambiguity (usually taken as an uncertainty inductor) and flexibility in their decision strategies [1,9].

Uncertainty has been traditionally associated with negative states such as fear and anxiety. However, evidence also links uncertainty to positive emotions like surprise, interest, excitement, and enthusiasm, depending on individuals’ expectations about a task [10]. Mood plays a significant role in decision-making and is associated with emotional states elicited by expectations, rewards, and uncertainty [11]. Mood in decision-making can be understood as a subjective experience and physiological changes occurring during task performance [12]. More specifically, mood relates to prolonged experiences marked by valence, motivation, and arousal [12,13]; positive mood is linked to creativity, originality, and flexibility [12]. The induction of mood varies between different experimental approaches, from using music or films to induce mood in participants [14,15] to the use of task incentives such as money [16]. Mood serves as a way to apprehend the participants’ emotional state when receiving rewards and losses. Within computational modeling, mood is operationalized as the cumulative effect of differences between reward outcomes and expectations [16]. It is thought to reflect the cumulative impact of several outcomes, as opposed to emotional reactions related typically to a single stimulus [17]. In our experimental design, mood is defined as the expected value of the prediction error history (see details below in Materials and Methods). We did not measure the subjective and physiological components of the emotional reaction of mood; instead, we used a computational construct that allow us to capture the influence of the appraisal of an agent’s action. Based on the operationalization of Bennett et al. [18], we propose that the valence of an agent’s mood corresponds to their appraisals of the difference between the value of an action and the value of the environmental state within which the action was taken. In this sense, the computational construct captures the moving average of surprise, that is, the history of how different the expectations and the real rewards are.

Emotions can shape how people learn from environmental cues by influencing cognitive processes such as confidence and metacognition. For instance, negative emotions can lead people to be more prone to risk-seeking behavior [19,20,21] opening the door to flexible and even creative solutions [10]. Recent research has focused on the influence of confidence on learning under uncertainty and how mood influences these executive processes [11]. Confidence, defined as the belief in the correctness of a decision, is associated with higher decision accuracy in learning tasks [22]. Confidence is typically measured by asking participants to judge their own decisions and then rate their confidence on a scale. Confidence rates can represent, altogether with the probability that a decision is correct, factors such as estimates of noise and evidence magnitude, among others. High confidence can be related to confirmation bias by affecting the post-decision processing of the integration of confirmatory and disconfirmatory evidence [23]. Furthermore, confidence involves prefrontal brain activity, which influences learning rates and social interactions, among others [22]. The relationship between mood and confidence has yet to be well explored, with some studies indicating that self-reported mood does not necessarily align with confidence as it evolves on different timescales [11].

Using a learning task (see Rollwage et al. [23], Doll et al. [24], Palminteri et al. [25]), we aimed to explore the relationship between confidence, mood, learning under uncertainty, and exploration–exploitation behavior. In our task, participants had to learn under uncertainty and under different reward states that induced different moods. We hypothesize that mood influences confidence, enabling higher decision flexibility (i.e., accurate switching between exploration and exploitation). This higher decision flexibility will lead to better task performance (higher accuracy). Our results show that, indeed, confidence modulates exploration/exploitation and learning rates, while mood modulates reward perception and confidence. These results suggest that the context of a decision can influence the strategies people use when facing uncertainty by modulating mood, which, in turn, affects their sensitivity to environmental changes and overall task performance. Specifically, we observed that a negative mood reduced confidence, leading to increased exploratory behavior. Conversely, a positive mood enhanced confidence, promoting a focus on exploitation and improving task performance. Taken together, our results suggest that metacognition involves a flexible balance between exploration and exploitation, integrating mood with high-level cognitive processes.

## 2. Materials and Methods

### 2.1. Participants

Twenty-two participants with normal or corrected-to-normal vision, all right-handed, performed an adaptation of a probabilistic learning task [23,24,25]. All subjects performed the same experimental task. Their ages ranged from 21 to 33 years (M = 26.3, SD = 4.46; 9 women). All participants provided written informed consent before inclusion in the study, following the Declaration of Helsinki [26]. The inclusion criteria required participants to be older than 18 years and to have no history of neurological or psychiatric disorders.

### 2.2. Experimental Task

The task was programmed using JsPsych 7.1.2, a JavaScript library designed for creating and running online experiments [27]. The experiment results were stored in a cloud-hosted NoSQL database using Google Firebase [28]. The Firebase Realtime Database, based on a JSON data structure, is well suited for storing complex data and supporting extensive applications. Database management and plot generation were implemented using the Python 3.10 programming language.

The experimental task consisted of 100 trials, each involving two decision points followed by a confidence rating for each. In each decision point, two stimuli (hiragana characters) were presented, with one offering a reward. Participants had to choose the stimulus they believed was more likely to provide a reward. They were not informed about the reward probabilities in advance, meaning they did not know how frequently each stimulus would offer a reward. Instead, they were instructed to learn through trial and error whether there were differences in the reward probabilities associated with each stimulus and which option was the best for obtaining a reward.

**Figure 1:**
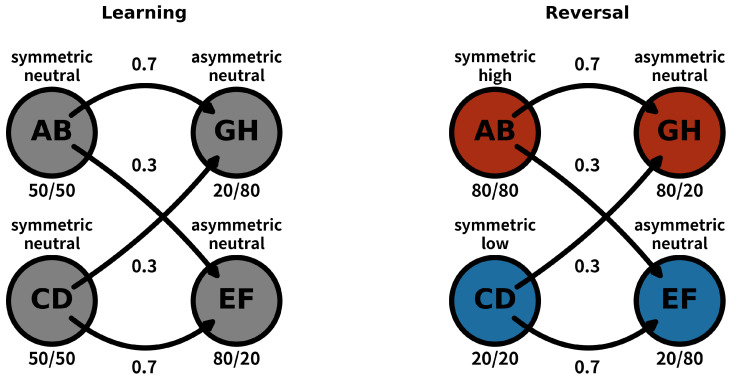
A scheme of the experimental task in its two different phases, learning and reversal.

**Figure 2:**
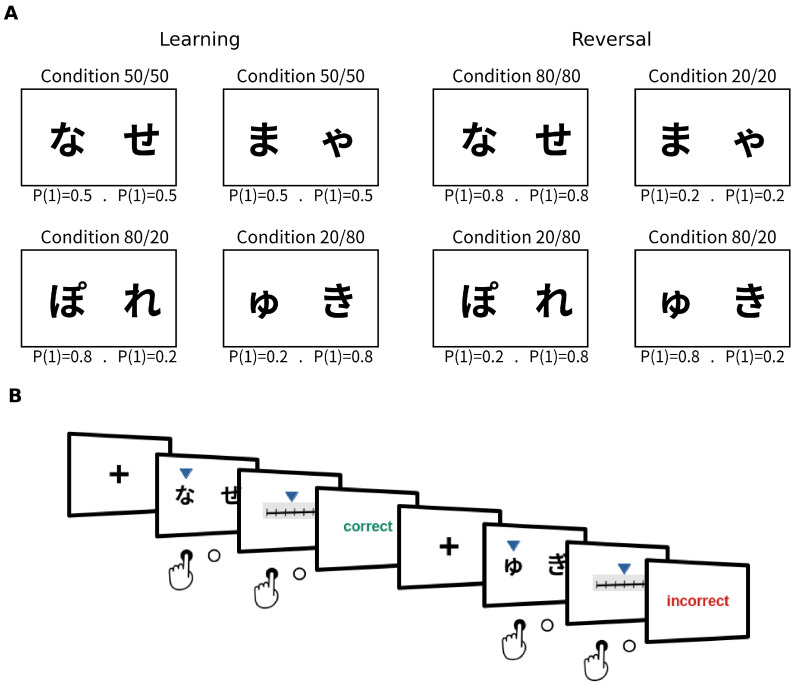
(**A**) Stimuli and probability distribution of rewards. (**B**) Typical trial of experimental task with positive and negative feedback.

In each trial, participants first encountered a decisions instance between two options with equal reward probabilities (a symmetric choice). They had to select one of the options and then rate their confidence in obtaining the reward on a scale from 1 to 10 (from low to high confidence). After this, participants faced a decision between two options with different reward probabilities (an asymmetric choice), where they again had to make a decision and rate their confidence in their response using the same scale. In summary, each trial consisted of two sequential decision-making instances, each followed by a confidence rating. In the task, we used Hiragana characters, in order to use a culturally neutral stimuli, but to enable easier understanding the task, we will use as a reference Latin alphabet letters for referring the the pairs. This setup resulted in the trial pairs AB-GH, AB-EF, CD-GH, and CD-EF, which were presented pseudo-randomly across both phases (see Figure 1 and Figure 2).

The experiment consisted of two phases, learning and reversal, with 50 trials each. During the learning phase, the symmetric pairs (AB and CD) had equal reward probabilities of 0.5. Given that the pairs AB and CD presented equal reward probabilities, we called these neutral reward states. The asymmetric pairs, on the other hand, provided biased positive feedback with the following probabilities: GH: 0.2/0.8; EF: 0.8/0.2 (see Figure 1). For instance, when participants were presented with the GH pair, by choosing the option H, they would have a 0.8 probability of obtaining a reward. These pairs facilitated a learning process in which participants needed to recognize the biased reward probabilities to improve task performance. During the reversal phase, the symmetric pair AB had a reward probability of 0.8, while the CD pair had a probability of 0.2. Due to the high reward probability of the AB pair, it was considered a high-reward state, whereas the CD pair represented a low-reward state. This created a new context for the asymmetric pairs, whose reward probabilities were reversed compared to the learning phase (see Figure 1). Thus, the beginning of each phase was considered to involve conditions of higher uncertainty (see Figure 1 and Figure 2).

In summary, the experimental protocol instructed participants to accumulate as many rewards as possible and informed them that some stimuli would lead to more frequent wins than others. Participants were not provided with explicit information about the reward probabilities, which they had to learn through trial and error, nor about changes in these probabilities. They were instructed to choose the stimulus they believed would offer a reward in each decision instance and to rate their confidence in their response (i.e., how certain they were about actually receiving the reward). Participants made their choices and rated their confidence by pressing a left or right arrow key with their right hand (choice timing was self-paced) (see Figure 2).

## 3. Behavioral Modeling

In this section, we delve into the nuances of the behavioral modeling through the lens of the Confidence Prediction-Error Momentum (CPEM) Model. This model elucidates the dynamic interplay between the expected values, rewards, and mood within decision-making processes by integrating parameters such as the confidence weighed (κ) on learning rate (α), choice stochasticity (β), and the mood-biased prediction error ($\delta_{t}^{\mathrm{pem}}$) (below, we provide a definition and operationalization of the parameters listed in Table 1). We construct a robust framework for understanding how cognitive and emotional states influence behavioral outcomes from a computational perspective.

The CPEM model offers a comprehensive approach to quantifying the latent values assigned to each action (a) and state (s) in a trial-by-trial analysis. Central to this model is the concept of prediction error, which captures the divergence between expected outcomes and actual rewards. An individual’s mood (μ) intricately modifies this error, introducing a unique layer of complexity to the reward perception and subsequent decision-making processes. The model dynamically updates the expected values and mood based on real-time interactions and outcomes by applying nonlinear functions and learning rates. This iterative learning process is pivotal in refining the accuracy of behavioral predictions under varying conditions of uncertainty and reward-state contexts. This section aims to provide a clear and detailed exposition of the Confidence Prediction-Error Momentum Model, emphasizing its potential applications and the theoretical underpinnings that make it a valuable tool for psychological and behavioral research.

We adjust the behavioral data to reinforcement learning models. The model space includes a standard Rescorla–Wagner model or Q-learning [29,30], from now on named RW; a modified version of the RW model that add mood as a measure of the recent history of prediction errors (named PEM) [31]; and 5 modified versions of the PEM model that add confidence monitoring of the influence of behavior and mood on confidence monitoring (named CPEM). In the next sections, we will explain the details of each model.

### 3.1. Basic Model-Free RL

We started with a basic, model-free reinforcement learning algorithm [30] (RW model). Trial-by-trial for each pair of stimuli (for instance, A and B), the model estimated the expected value, based on the individual results history, and used this expected value ($Q_{s,a,t}$) for making a decision (for instance, taking an action *a* for A instead of B in state *s*). The expected values were zero before learning, and after each trial *t*, the choices’ estimated value was updated as a function of the prediction error. This update of the value is the learning rate, and the updating follows the delta rule [29,30]:

(1) $$\begin{array}{cc} Q_{s,a,t+1} & =Q_{s,a,t}+\alpha \delta_{t} \end{array}$$

and we keep the expected value for the non-chosen option, where “¬a” represents the absence of the action a, in other words, the non-chosen option:

(2) $$\begin{array}{cc} Q_{s,\neg a,t+1} & =Q_{s,\neg a,t} \end{array}$$

where $\alpha_{t}$ is the learning rate (the updated value) and $\delta_{t}$ is the prediction error defined as the difference between the actual reward ($r_{t}$) and expected value ($Q_{s,a,t}$):

(3) $$\begin{array}{cc} \delta_{t} & =r_{t}-Q_{s,a,t} \end{array}$$

$\alpha_{t}$ and $\delta_{t}$ update their value in Equation (1), and are not present in the non-chosen choice (Equation (2)) given that this does not present information to be updated.

### 3.2. Mood Effects on Valuation

In order to include the mood effect in the valuation of rewards, Eldar and Niv [31] include the perceived rewards in the calculation of the prediction error, instead of the real rewards:

(4) $$\begin{array}{cc} r_{t}^{\mathrm{pem}} & =r_{t}\gamma^{\mu_{t}} \end{array}$$

(5) $$\begin{array}{cc} \delta_{t}^{\mathrm{pem}} & =r_{t}^{\mathrm{pem}}-Q_{s,a,t} \end{array}$$

Here, $r_{t}^{\mathrm{pem}}$ represents the bias effect of mood over rewards; thus, $\mu_{t}$ indicates a positive mood ($0<\mu_{t}<1$) or a negative mood ($-1<\mu_{t}<0$), and γ is a constant parameter that shows the direction and scope of mood, restricted to the interval 0<γ≤3 in this study. If γ>1, mood exerts positive feedback, as reward is perceived as larger in a good mood and as smaller in a bad mood. Conversely, 0<γ<1 corresponds to negative feedback, as reward is perceived as smaller under a positive mood and as larger under a negative mood. If γ=1, mood does not bias the perception of rewards Eldar and Niv [31]. Eldar and Niv [31] modeled the effects of unexpected results over mood, assuming that mood represents a recent history of prediction errors (μ) independent of state or context (Prediction Error Mood Model, from now on named PEM), restricted to the interval ($-1<\mu_{t}<1$) by means of a hyperbolic tangent function:

(6) $$\begin{array}{cc} \mu_{t+1} & =\mu_{t}+\eta \left(r_{t}-Q_{s,a,t}-\mu_{t}\right) \end{array}$$

(7) $$\begin{array}{cc} \mu_{t} & =\tanh(\mu_{t}) \end{array}$$

where η is a constant learning rate (mood update), restricted to the interval 0≤η≤1. Following th RW model, the prediction error $\delta_{t}^{\mathrm{pem}}$ updates the expected values after each trial with a learning rate of $\alpha_{t}$:

(8) $$\begin{array}{cc} Q_{s,a,t+1} & =Q_{s,a,t}+\alpha_{t}\delta_{t}^{\mathrm{pem}} \end{array}$$

(9) $$\begin{array}{cc} Q_{s,\neg a,t+1} & =Q_{s,\neg a,t} \end{array}$$

### 3.3. Confidence Monitoring

Regardless of the CPEM variants, we estimated the expected confidence value trial-by-trial ($C_{s,t+1}$), adding different predictors used in previous studies that use a learning task with two forced options (2AFC) [32,33,34,35]. For instance, Lebreton et al. [33] and Somatori and Kunisato [34] modeled confidence, adding as a regressor the difference in the evidence for each option in the decision time, which reflects the difficulty of distinguishing the best choice. This, in an RL model can be operationalized as the absolute value of the expected value difference for a contingent pair of options ($|{\Delta Q}_{s,t}|$).

(10) $$\begin{array}{cc} C_{s,t+1} & =C_{s,t}+\left|{\Delta Q}_{s,t}\right|+\theta_{t}\delta_{t}^{C} \end{array}$$

where $\delta_{t}^{C}$ is a delta rule that updates the confidence values from the prediction errors [32,35], with a confidence learning rate of $\theta_{t}$, restricted to the interval $0\leq \theta_{t}\leq 1$:

(11) $$\begin{array}{cc} \delta_{t}^{C} & =\delta_{t}^{\mathrm{pem}}-C_{s,t} \end{array}$$

### 3.4. Meta-Learning Modulation

The estimated confidence was used for modeling the free parameters in the reinforcement learning models. This was carried out after each result, providing information about the precision of the reinforcement learning model regarding the value estimations and the behavioral strategies. The reinforcement models have constant parameters: learning rate (parameter $\alpha_{t}$) and choice stochasticity (which has been linked to exploration and exploitation; for more details, see below subsections “Confidence Modulation on Choice Stochasticity” and “Choice probability”). This limits the capacity for optimizing the behavioral policy (the strategy) at the end of the learning blocks once subjects believe that they have a reasonably good estimation of contingencies. At this point, the prediction errors have to be moderated and the choices have to be adjusted to a more deterministic exploitation of the learned contingencies [36,37,38]. On the contrary, when contingencies change (at the beginning of the reversal phase), prediction errors should weigh more and choices should be more exploratory. A method of optimizing behavior is to subordinate the reward-learning parameters to a higher control level that monitors the performance. Thus, the CPEM models included a metacognitive level that updated confidence to regulate contingency learning and stochasticity of choice.

### 3.5. Confidence’s Modulation of Learning Rate

Confidence can modulate the learning rate. Lower confidence means higher learning rates because the knowledge of the environment is lower [39,40]. Higher confidence has the opposite effect; the learning rates should be lower since a decision-maker that trusts in their decisions does not need to update their value estimations. Learning rates were modulated based on confidence and by asymmetrically inducing confirmation bias, that is, when the expected value of the option and the valence of the observed reward match (positive bias) [25,41].

Instead of estimating two different learning rates ($\alpha^{-}$ and $\alpha^{+}$), increasing the model complexity, we defined a constant $w_{\alpha }=4$ if the result is confirmatory; otherwise, $w_{\alpha }=1$, which increases the modulating effect of κ (weight of confidence) in the dynamic learning rate $\alpha_{t}$, inducing asymmetry as follows:

(12) $$\begin{array}{cc} \alpha_{t} & =\frac{\alpha }{1+w_{\alpha }\kappa C_{s,t}} \end{array}$$

where α is the learning rate value when the effect of the estimated confidence value is absent. Moreover, $\alpha_{t}$ is the dynamic coefficient of the learning rate (models CPEM, CPEM_1_, and CPEM_3_)and $\alpha_{t}=\alpha$ if it is constant (models RW, PEM, CPEM_0_, and CPEM_2_).

**Figure 3:**
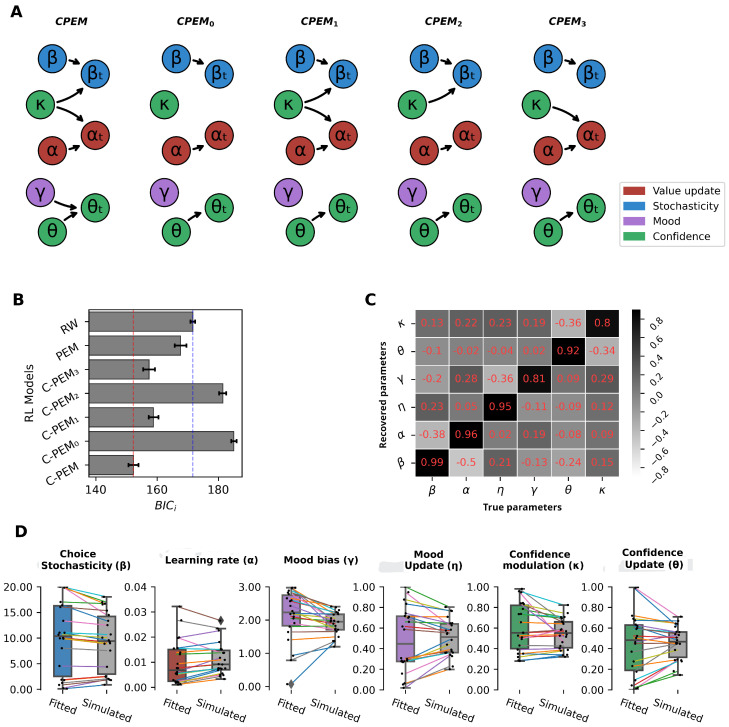
(**A**) Parameter space. Parameter space for CPEM (best model) and other CPEM candidates (0 to 3). (**B**) Model comparison. Comparison of goodness-of-fit of seven RL models: Confidence and Prediction-Error Mood Model (CPEM and other CPEM candidates (0 to 3)), Prediction Error Momentum (PEM) model, and classical Rescorla–Wagner (RW) model. Plotted is average Bayesian Information Criterion (BIC) for subjects. Smaller BIC values indicate better fits. (**C**) Parameter recovery. Pearson correlation coefficients between model parameters used to generate behavior (true parameters), and their recovered counterparts (recovered parameters). (**D**) Model simulations. CPEM parameters fitted to data (fitted) and average parameter simulations (simulated; N simulations = 500). CPEM Model simulations were obtained using individual best fitting free parameters.

The selection of the constant $w_{\alpha }$ follows three criteria: Firstly, the empirical value of the asymmetry in the learning rates of previous studies suggest that the $\alpha^{-}$ value is close to a 30% of $\alpha^{+}$ [25,41,42,43], which is possible for $w_{\alpha }$≈ 4 values. Secondly, we conduct sensitivity analyses testing different confirmatory constant values for the CPEM model. Thirdly, we consider the alternative of making $w_{\alpha }$ an additional parameter, increasing the model complexity (see Figure 3B for the model comparison, and Supplementary Materials, Figure 1, to see an extensive comparison).

### 3.6. Confidence’s Modulation of Choice Stochasticity

Similarly, confidence can modulate global behavioral variables such as choice stochasticity, which refers to how deterministic or random the decision strategies were. There is evidence showing that uncertainty influences exploration, since lower confidence is used to estimate the value for signaling a change over a more exploratory strategy [44]. Dynamic choice stochasticity $\beta_{s,t}$, that is, the randomness of decisions throughout the task, was modulated in such a way that state-dependent exploration for the next trial was reduced when the present estimated confidence increased:

(13) $$\begin{array}{cc} \beta_{s,t+1} & =\beta_{ub}-\frac{\beta_{ub}-\beta }{1+2\kappa C_{s,t}} \end{array}$$

where $\beta_{ub}=20$ is the upper bound for parameter β, which is the choice stochasticity value when the effect of confidence is absent, and κ, which is the weight of confidence on choice stochasticity $\beta_{s,t}$.

### 3.7. Mood’s Modulation of Confidence Update

Within all CPEM model variants, the updating of the estimated confidence was controlled by the delta rule $\delta_{t}^{C}$ and the parameter $\theta_{t}$. However, only for the saturated CPEM model was the mood modulation included $\gamma^{\mu_{t}}$ for $\theta_{t}$:

(14) $$\begin{array}{cc} \theta_{t} & =\sigma \left(\frac{\theta }{\gamma^{\mu_{t}}}\right) \end{array}$$

where θ is the confidence learning rate without the mood effect and $\theta_{t}$ was escalated to the range ($0\leq \theta_{t}\leq 1$) with a sigmoidal function:

(15) $$\begin{array}{c} \sigma \left(x\right)=\frac{1}{1+e^{x}} \end{array}$$

For models CPEM_0−3_, $\theta_{t}=\theta$ is constant.

### 3.8. Choice Probability

The probability of accuracy was estimated from the choices’ expected value differences with respect to the sigmoidal function:

(16) $$\begin{array}{cc} p_{s,t}\left(\mathrm{right}\right) & =\frac{1}{1+e^{-\beta_{s,t}\left(Q_{s,t}\left(\mathrm{right}\right)-Q_{s,t}\left(\mathrm{left}\right)\right)}} \end{array}$$

where $\beta_{s,t}$ is the dynamic choice stochasticity coefficient (models CPEM, CPEM_1_, snd CPEM_2_) and $\beta_{s,t}=\beta$ if it is constant (models RW, PEM, CPEM_0_, and CPEM_3_).

### 3.9. CPEM Model Space

The construction of the CPEM models builds on the PEM model, adding an additional layer of complexity by incorporating confidence and its interaction with mood, value updating, and choice stochasticity. The various specifications of the CPEM models account for different computational hypotheses regarding the modulation and dynamics of value updating (learning rate) and choice stochasticity by mood and confidence (see Figure 3A).

The CPEM model (best model) considers that the value update and choice stochasticity are dynamics ($\alpha_{t}$ and $\beta_{t}$, respectively); this means they are updated trial-by-trial by confidence (parameter κ, nodes $\kappa \to \alpha_{t}$ and $\kappa \to \beta_{t}$ in Figure 3A). Moreover, parameter α, node $\alpha \to \alpha_{t}$ and parameter β, node $\beta \to \beta_{t}$ are idiosyncratic (non-dynamic) for each subject (see Equations (12) and (13), respectively). Additionally, the model considers that confidence updating ($\theta_{t}$) is dynamically modulated by mood (parameter γ, node $\gamma \to \theta_{t}$ in Figure 3A) and by the idiosyncratic confidence learning rate of each subject (parameter θ, node $\theta \to \theta_{t}$ in Figure 3) (see Equation (14)).

The alternative CPEM models (CPEM_0,1,2,3_) start from the CPEM model, but can omit some of the specific dynamic modulations. The CPEM_0_ model (see Figure 3A) omits the dynamic modulation of confidence (parameter κ) over the value update and choice stochasticity (nodes $\kappa ↛\alpha_{t}$ and $\kappa ↛\beta_{t}$ in Figure 3A), reducing the modulation to the idiosyncratic parameters (parameters α and β, nodes $\alpha \to \alpha_{t}$ and $\beta \to \beta_{t}$ in Figure 3A). Thus, Equations (12) and (13) are simplified to $\alpha_{t}=\alpha$ and $\beta_{t}=\beta$, respectively. In the same way, the absence of the dynamic modulation of mood over confidence update (node $\gamma ↛\theta_{t}$ in Figure 3A) reduces the relation $\theta \to \theta_{t}$, and simplifies Equation (14) to $\theta_{t}=\theta$.

The CPEM_1_ model only omits the dynamic modulation of the confidence update by mood (node $\gamma ↛\theta_{t}$ in Figure 3A), and reduces the relation $\theta \to \theta_{t}$, and simplifies Equation (14) to $\theta_{t}=\theta$.

The CPEM_2_ and CPEM_3_ models omit the dynamic modulation of confidence both over the value update (CPEM_2_, node $\kappa ↛\alpha_{t}$ in Figure 3A) and over the choice stochasticity (CPEM_3_, node $\kappa ↛\beta_{t}$ in Figure 3A), respectively. Both models CPEM_2_ and CPEM_3_ also omit the dynamic modulation of the confidence update by mood (node $\gamma ↛\theta_{t}$ in Figure 3A), reducing the relation to $\theta \to \theta_{t}$ and simplifying Equation (14) to $\theta_{t}=\theta$.

## 4. Statistical Analysis

All the statistical analyses, that is, the computational modeling and the statistical tests (parametric and non-parametric), the cluster test permutation, and the linear mixed model (LMM), were implemented in Python 3.10.

### 4.1. Model Fitting

For the computational modeling, a maximum likelihood approach was used to estimate the parameter values that best fit the behavioral data. This approach seeks the parameter values of the model that maximize the likelihood of the data given the parameters. Maximizing the likelihood is equivalent to maximizing the log-likelihood (or minimizing the negative log-likelihood), which can be expressed in terms of the choice probabilities of the individual model.

The optimization procedure to find the global and local minima of the negative log-likelihood function was implemented complementarily with the *optimize.differentiale_evolution* and *optimize.minimize* functions from the *SciPy 7.1.2* module, applied iteratively (500 iterations per subject × model). Parameter bounds were defined based on values reported in the literature (see Table 1). For each candidate model and subject, we calculated the Bayesian Information Criterion (BIC), reducing the maximum likelihood by the number of free parameters in the model.

The random-effects BIC analysis showed that the CPEM model more accurately represented the data compared to the RW model (BIC_RW_ = 171.66 ± 0.94, BIC_CPEM_ = 152.23 ± 1.97; mean ± SEM) and the PEM model (BIC_PEM_ = 167.62 ± 2.30, BIC_CPEM_ = 152.23 ± 1.97), even accounting for its additional degrees of freedom (see Figure 3B for the comparison of the main candidate models and Supplementary Figure S1 for the comparison of all evaluated models).

To validate the inference procedure, we performed parameter recovery by taking the model’s estimated parameters for each subject as the true parameters (true parameters in Figure 3C and fitted in Figure 3D) and simulated behavioral data based on them (simulated in Figure 3D). We then estimated the parameters from the simulated data (recovered parameters in Figure 3C) and compared them to the true parameters. The correlation between the true and recovered parameters was generally high (r ≥ 0.8; see Figure 3C).

### 4.2. Transformed Variables

Metacognitive bias was measured as the trial-by-trial difference between choice probability and sigmoid scale confidence values estimated by the best CPEM model:

(17) $$\begin{array}{cc} \mathrm{Metacognitive}\;{\mathrm{bias}}_{s,t} & =\mathrm{Choice}\;{\mathrm{Probability}}_{s,t}-\sigma {\left(\mathrm{Confidence}\;\mathrm{Value}\right)}_{s,t} \end{array}$$

The coefficients modulated by confidence (dynamic choice stochasticity $\beta_{t}$ and dynamic learning rate or value update $\alpha_{t}$; for details, see below behavioral modeling) were divided additively in a fixed part (free parameters β and α) and a variable one:

(18) $$\begin{array}{cc} \beta_{t} & =\beta +\beta_{C}\to \beta_{C}=\beta_{t}-\beta \end{array}$$

(19) $$\begin{array}{cc} \alpha_{t} & =\alpha +\alpha_{C}\to \alpha_{t}-\alpha \end{array}$$

The variable component was derived from the dynamic coefficients, which were attributed to modulation confidence, value updates ($\alpha_{C}$), and choice stochasticity ($\beta_{C}$). Variables exhibiting biases in their distributions were transformed to maintain zero as the reference and preserve the sign, using the signed square root transformation ($\mathrm{sign}\left(x\right)\;\cdot \;\sqrt{\left|x\right|}$).

## 5. Results

### 5.1. Mood’s Modulation of Reward and Choice

**Figure 4:**
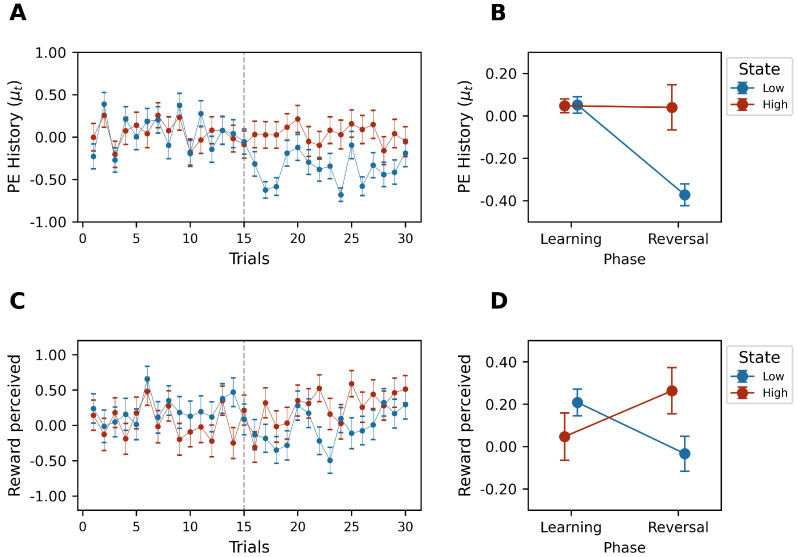

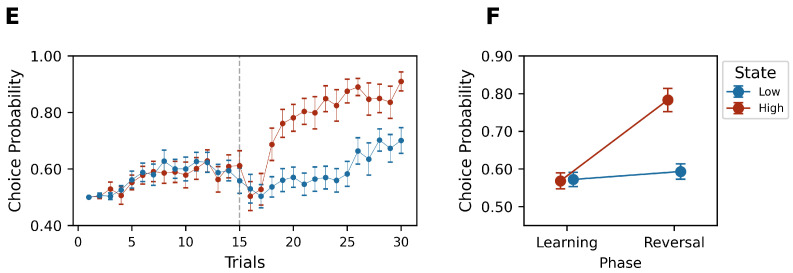
(**A**,**C**,**E**) Trial–by–trial group prediction error history, reward perceived, and choice probability average, respectively, by high (red)– and low (blue)–reward states. (**B**,**D**,**F**) Estimated mean of prediction error history, reward perceived, and choice probability, respectively, of Phase × State interaction effect (red: high-reward state; blue: low-reward state). Error bars represent standard error of mean (SEM).

This research investigated the dynamics of mood, confidence, and decision-making strategies under varying reward conditions. Our findings illustrate the significant modulation of mood (defined as the expected value of the prediction error history) by the experimental design (Phase×State:F(1,21)=21.594, p< 0.05, $\eta_{p}^{2}=0.507$), particularly during the reversal phase, where mood estimations were markedly higher in the high-reward state than the low-reward state (W=23.0,p< 0.05, ${\mathrm{Cohen}}^{\prime }s\;d=1.149$) (see Figure 4A,B).

The temporal flow shown in Figure 4A illustrates the significant drop in mood when the decision environment changed (at the beginning of the reversal phase) in the low-reward state. In line with this result, the perceived rewards were significantly higher ($\mathrm{Phase}\times \mathrm{State}:F\left(1,21\right)=16.154,\;p<0.05,\eta_{p}^{2}=0.435$) during the high-reward state ($W=40.0,\;p<0.05,\;{\mathrm{Cohen}}^{\prime }s\;d=0.979$), showing the influence of mood on reward perception (see Figure 4C,D).

Moreover, during the learning phase, there were no differences between the accuracy values (defined as the probability of the assignment of a higher predicted value) (W=112.0, p=0.656, ${\mathrm{Cohen}}^{\prime }s\;d=-0.043$), but during the reversal phase, there was a higher proportion of choices with higher predicted values in the high-reward state ($\mathrm{Phase}\times \mathrm{State}:F\left(1,21\right)=25.612,\;p<0.05,\eta_{p}^{2}=0.549$) ($W=15.0,p<0.05,\;{\mathrm{Cohen}}^{\prime }s\;d=1.707$) (see Figure 4E,F).

### 5.2. Confidence Monitoring

**Figure 5:**
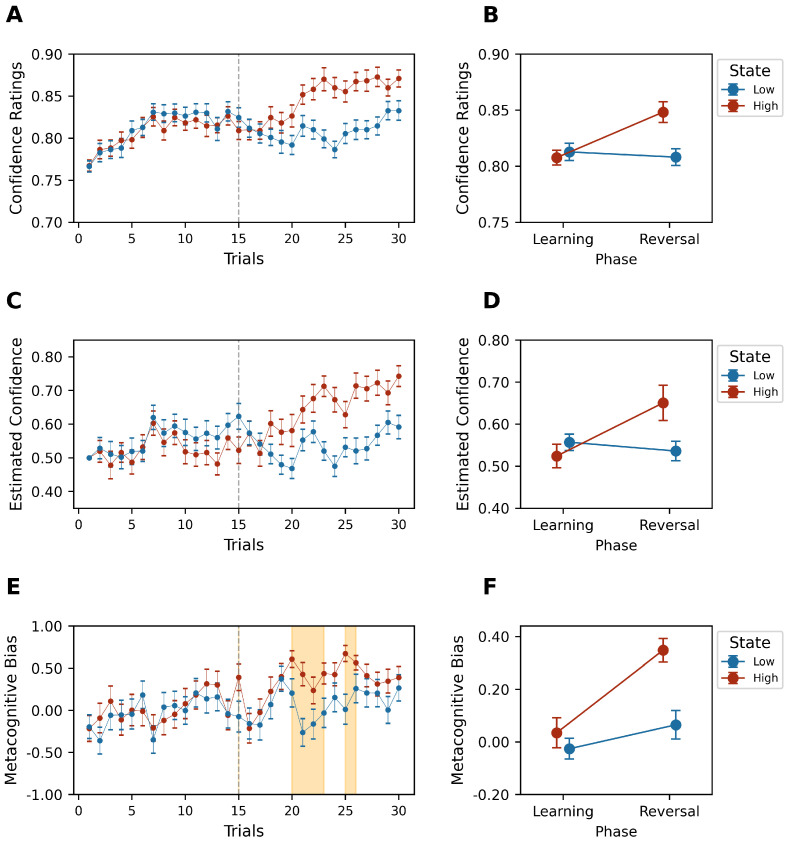
(**A**,**C**,**E**) Trial–by–trial group confidence ratings, estimated confidence, and metacognitive bias average, respectively, by high (red)– and low (blue)– reward states. The orange area corresponds to the temporary clusters identified by the cluster-based permutation Mixed ANOVA test (threshold p<0.05, 10,000 permutations). (**B**,**D**,**F**) Estimated mean of confidence ratings, estimated confidence, and metacognitive bias, respectively, of Phase × State interaction effect (red: high-reward state; blue: low-reward state). Error bars represent SEM.

The analysis revealed that confidence levels were consistently elevated in the high-reward state across the reversal phase experiment phases (Phase×State: F(1,21)=23.819, $p<0.05,\eta_{p}^{2}=0.531$) ($W=12.5,p<0.05,\;{\mathrm{Cohen}}^{\prime }s\;d=1.123$) despite no notable differences observed during the learning phase ($W=82.0,p=0.251,\;{\mathrm{Cohen}}^{\prime }s\;d=-0.165$) (see Figure 5A,B). This pattern underscores a robust association between reward conditions and confidence ratings, with higher rewards fostering greater confidence.

To quantify the quality of the confidence value estimates from the best CPEM model, we fitted a linear mixed-effects model (LMM) to predict confidence ratings based on the estimated confidence values (both standardized using z-scores). The model specification included random effects to account for variability between subjects, capturing each subject’s deviation from the overall intercept and slope in relation to each trial:

(20) $$\begin{array}{cc} \mathrm{Confidence}\;{\mathrm{Ratings}}_{ij} & =\beta_{0}+\beta_{1}\mathrm{Confidence}\;{\mathrm{Estimated}}_{ij}+b_{0i}+b_{1i}{\mathrm{Trial}}_{ij}+\epsilon_{ij} \end{array}$$

Thus, the fitted model demonstrates that the confidence value estimates from the best CPEM model achieve a good fit with the empirical confidence data (β=0.60, SE=0.01, t=34.06,p<0.001), explaining 63% of the variance in confidence ratings (conditional $R^{2}=0.63$/marginal $R^{2}=0.36$) (AIC=2566.02,BIC=2597.13).

The confidence estimated by the model has the same shape, showing the same effect ($\mathrm{Phase}\times \mathrm{State}:F\left(1,21\right)=28.869,p<0.05,\eta_{p}^{2}=0.579$) (W=8.0,p<0.05, ${\mathrm{Cohen}}^{\prime }s\;d=1.518$) (see Figure 5C,D).

Moreover, our results indicated that the high-reward state enhanced confidence and improved metacognitive bias ($\mathrm{Phase}\times \mathrm{State}:F\left(1,21\right)=5.192,p<0.05,\eta_{p}^{2}=0.198$) (see Figure 5E,F). This is interesting given the higher proportion of correct choices and confidence ratings observed during the high-reward state in the reversal phase (W=29.0, $p<0.05,\;{\mathrm{Cohen}}^{\prime }s\;d=1.211$). This was confirmed by the cluster (cluster mass statistic = 40.60, p<0.001)

### 5.3. Parameter Dynamics

**Figure 6:**
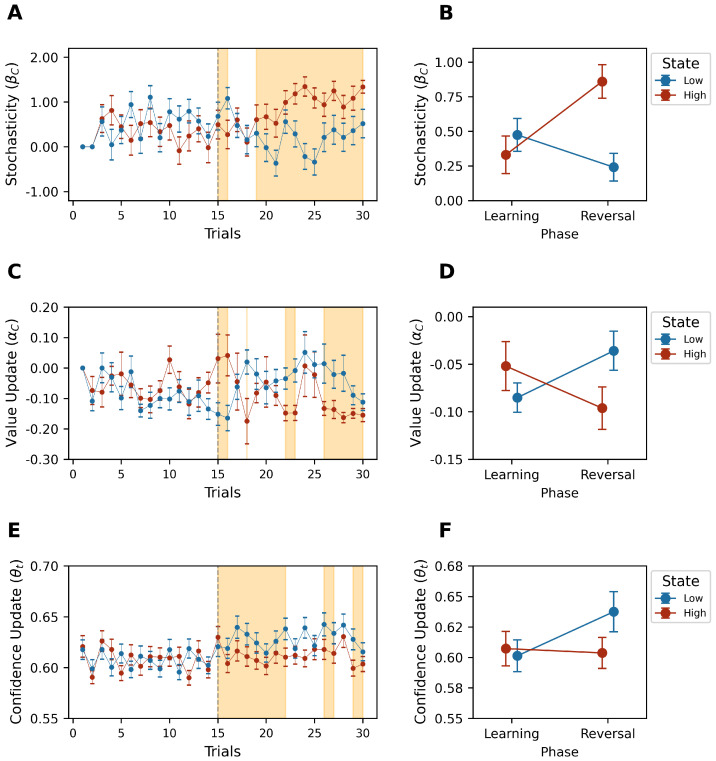
(**A**,**C**,**E**) Trial–by–trial group stochasticity, value update, and confidence update average, respectively, by high (red)– and low (blue)–reward states. The orange area corresponds to the temporary clusters identified by the cluster-based permutation Mixed ANOVA test (threshold p<0.05, 10,000 permutations). (**B**,**D**,**F**) Estimated mean of stochasticity, value update, and confidence update, respectively, of Phase × State interaction effect (red: high-reward state; blue: low-reward state). Error bars represent SEM.

This study also explored the effects of confidence on decision-making strategies. In conditions of high confidence, participants demonstrated a pronounced tendency to exploit the known options (higher stochasticity ($\beta_{C}$)), aligning with our hypothesis that confidence can significantly sway decision-making towards exploitative strategies ($\mathrm{Phase}\times \mathrm{State}:F\left(1,21\right)=13.438,p<0.05,\eta_{p}^{2}=0.39$) ($W=8.0,p<0.05,\;{\mathrm{Cohen}}^{\prime }s\;d=1.188$) (see Figure 6A,B). This was confirmed by the cluster (cluster mass statistic = 152.55, p<0.001).

Conversely, the low-reward state, characterized by more frequent value updates ($\alpha_{C}$) ($\mathrm{Phase}\times \mathrm{State}:F\left(1,21\right)=11.464,p<0.05,\eta_{p}^{2}=0.353$) ($W=53.0,p<0.05,\;{\mathrm{Cohen}}^{\prime }s\;d=-0.839$) and lower confidence, showed an increased propensity for exploration, suggesting a link between less confidence and a greater openness to exploring new options (see Figure 6C,D). This was confirmed by the cluster (cluster mass statistic = 56.281483, p<0.001).

Moreover, a negative mood was associated with a higher confidence update ($\theta_{t}$) ($\mathrm{Phase}\times \mathrm{State}:F\left(1,21\right)=5.616,p<0.05,\eta_{p}^{2}=0.211$) ($W=37.0,p<0.05,\;{\mathrm{Cohen}}^{\prime }s\;d=-0.391$). This was confirmed by the cluster (cluster mass statistic = 74.199993, p<0.001) (see Figure 6E,F).

### 5.4. Choice Entropy

**Figure 7:**
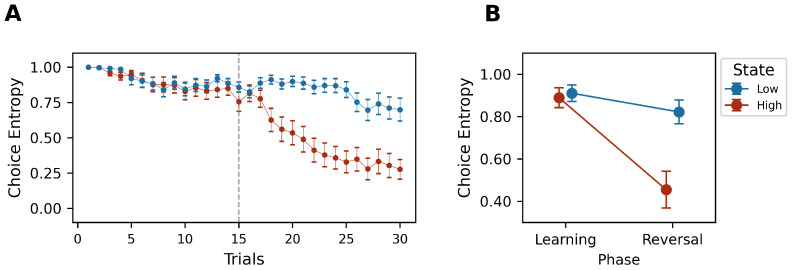
(**A**) Trial-by-trial group entropy average by high (red)- and low (blue)-reward states. (**B**) Estimated mean of entropy of Phase × State interaction effect (red: high-reward state; blue: low-reward state). Error bars represent SEM.

In line with previous results, the entropy of choice probability decreased progressively throughout the task, with notably lower levels during high-reward states ($\mathrm{Phase}\times \mathrm{State}:F\left(1,21\right)=25.27,p<0.05,\eta_{p}^{2}=0.546$) ($W=17.0,p<0.05,\;{\mathrm{Cohen}}^{\prime }s\;d=-1.61$). This finding indicates a clear shift toward more directed and exploitative strategies in high-reward conditions, compared to a slower change from the randomized exploration observed in low-reward states (see Figure 7A,B).

These insights collectively highlight the complex interplay between reward conditions, mood, and cognitive processes in shaping human decision-making, offering valuable implications for understanding the psychological drivers of behavior in uncertain environments.

## 6. Discussion

In this study, we hypothesized that mood influences confidence, enabling higher decision flexibility for accurate switching between exploration and exploitation decision strategies under uncertainty. Our results suggest that reward states influence mood estimations as expected. We observed that the reward states importantly influenced accuracy, confidence, and the updating process of choice values. This suggests that contexts with higher rewards facilitate learning under uncertainty. The difference in the learning rates is associated with higher confidence rates, which influence exploitation, during the reversal phase. Moreover, during the low-reward state, subjects updated the choice’s values more frequently, being more sensitive to the trial-by-trial nature of the test. This suggests that mood might influence the flexibility to explore new strategies and exploit new learning.

Recently, an interesting distinction has been proposed between directed exploration and random exploration, nuancing the dichotomy between exploiting and exploring alternatives when deciding. Directed exploration refers to establishing a goal and is driven by information (or identification of patterns), while random exploration is driven by chance [45]. Evidence has shown a relationship between long-term thinking (operationalized as temporal discounting) and directed exploration, associated with the long-term benefit of this strategy compared to exploiting known alternatives [5]. In line with this idea, mood might influence how participants read environmental cues in critical moments when exploring new strategies and facilitate the metacognitive integration of signaling for “closing” the exploration time to benefit from what was recently learned. This is also related to the metacognitive effect that was observed between the different reward states, in that metacognition was high during the high-reward context in which confidence had a markedly stronger influence on exploitation. There is evidence showing drops in confidence following errors in a decision task (even without external feedback) [46,47,48], and confidence, in turn, has been related to explorative strategies [49]. For instance, in a perceptual manipulation study Desender et al. [49] found that participants were more prone to seeking additional information when they were less confident in their decisions. Moreover, the opposite relation has been also established. That is, boosting confidence makes subjects less prone to seeking alternative information in searching tasks [50]. It is notable that there is evidence relating negative mood with lower metacognition even when this has no impact on decision accuracy [51].

The differences between the confidence rates in our experiment are consistent with the previous evidence. Participants were more confident in the high-reward state, which influenced the exploitation of the choice with higher expected value. Although overconfidence can be detrimental in specific decision contexts, in this case, higher confidence rates positively influenced the accuracy of the strategy during the reversal phase. The reversal phase presented to participants a sudden change in their decision environment through the two new reward-state contexts and inversion of the reward probability. This new environment, however, was again stable until the end of the task. In this sense, the cognitive challenge involved correct reading of the change, but also stability afterward. In decision terms, this involves correct exploration of new alternatives, and the exploitation of what was learned. This makes sense with the relation between higher confidence and less exploration previously mentioned, and with the influence of confidence on post-decision processing, biasing the weight of environmental cues over the expected results [23]. We can hypothesize that positive mood, in our case in the form of a higher expected value of the prediction error history of the task, modulates how the changes in the decision environment elicit metacognitive processing, which changes the updating frequency of present values, opening the door to exploration, which quickly turns into a newly defined strategy that is then exploited as confidence grows.

Our results, as we previously mentioned, collectively highlight the complex interplay between reward conditions, mood, and cognitive processes in shaping human decision-making. They suggest that different reward states as mood inductors influence learning under uncertainty. This influence occurs through integration of the history of rewards and the reading of the present environment. Mood, taken as a computational construct in our study, modulates the weight of rewards in behavioral planning, making them more salient in critical moments. Metacognition allows the detection of these critical moments, which might be the key to the correct adaptation to environmental changes in uncertain contexts.

As highlighted by one of the anonymous referees, it is important to acknowledge that these results face limitations associated with the lack of physiological measures of emotional states and a small sample of subjects with no variability in sociodemographic characteristics. Incorporating physiological and subjective measures of mood could provide more definitive evidence regarding the role of emotions in decision-making under uncertainty. These additions may also shed light on individual differences or temporal factors influencing the variables under study. For instance, such measures could help explain why certain participants appear more sensitive to trial-by-trial or historical conditions within the task while also offering deeper insights into other factors, such as attention or fatigue, that may affect decision-making processes.

Moreover, environmental changes necessitate distinct cognitive dynamics for adapting decision strategies, potentially influenced by personality traits. Future research should address these gaps by including additional measures of emotional states, assessments of personality traits, and clinical samples, as well as employing more extensive and more diverse participant groups. Such steps would enhance the precision and applicability of the proposed cognitive models, enabling a more comprehensive understanding of the mechanisms underlying adaptive decision-making in uncertain contexts and yielding more robust and generalizable results.

## Figures and Tables

**Table 1:** Free parameter and dynamic variable descriptions from RL models.

Parameters	
β	Choice stochasticity when estimated confidence = 0 ( 0<β≤20 ).
α	Learning rate when estimated confidence = 0 ( 0≤α≤1 ).
η	Mood-learning rate ( 0≤η≤1 ).
γ	Mood bias on reward and confidence update ( 0<γ≤3 ).
θ	Confidence learning rate ( 0≤θ≤1 ).
κ	Confidence modulation on learning rate and choice stochasticity ( 0≤κ≤1 ).
Dynamic Variables	
$\beta_{t}$	Dynamic choice stochasticity (scaled at 0<β≤20 ).
$\alpha_{t}$	Dynamic learning rate (scaled at 0≤α≤1 ).
$\theta_{t}$	Dynamic confidence learning rate (scaled at 0≤θ≤1 ).

## Data Availability

The data used for this study are available upon reasonable request.

## Supplementary Materials

The following supporting information can be downloaded at: https://www.mdpi.com/article/10.3390/bs14121144/s1, Figure S1: Comparison of all evaluated models.
