# Supplementary figures and images for: Navigating Uncertainty: The Role of Mood and Confidence in Decision-Making Flexibility and Performance
Source: Behav Sci (Basel). 2024 Nov 28;14(12):1144. doi: 10.3390/bs14121144 (PMC11673058; doi:10.3390/bs14121144)

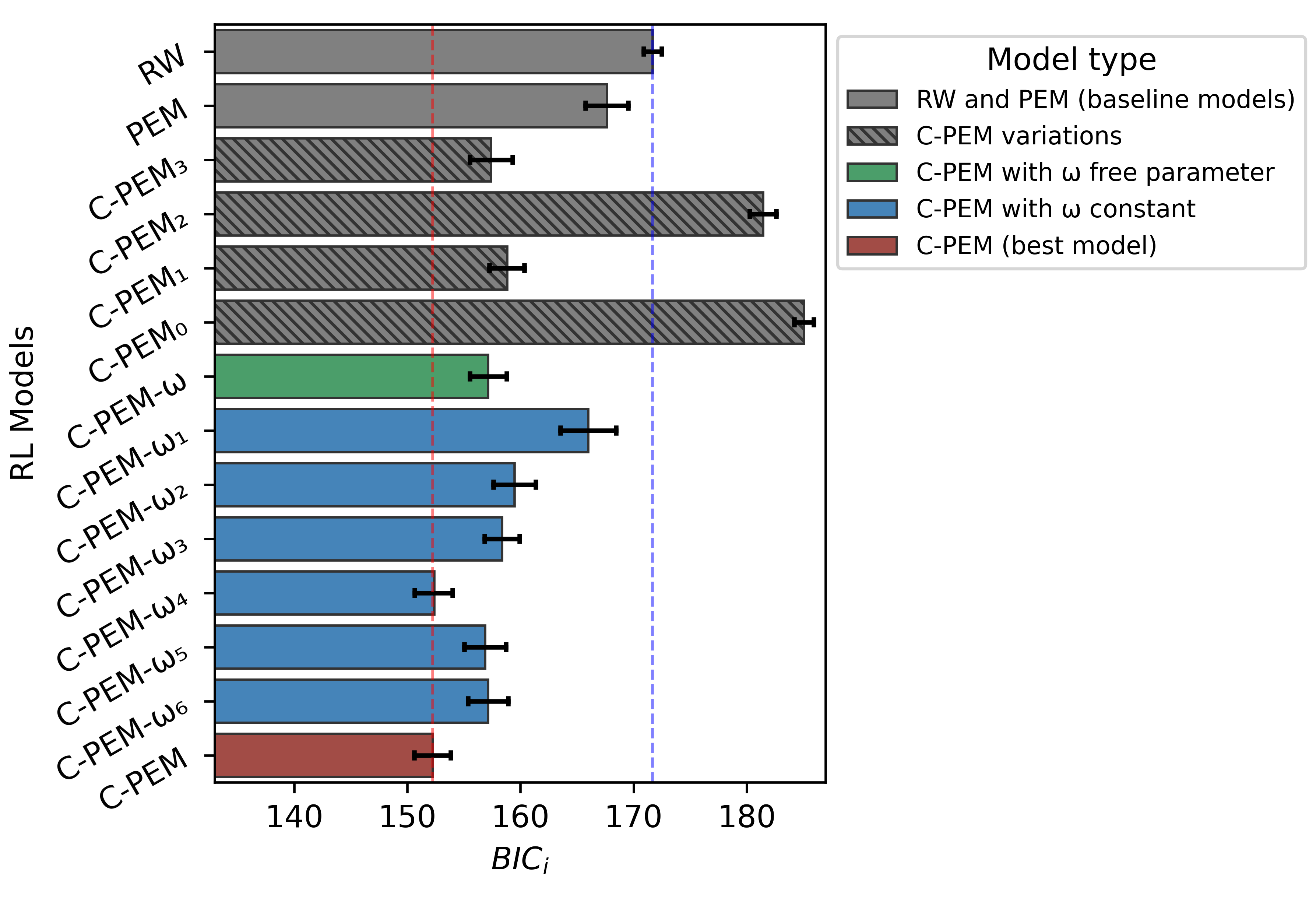

Supplement: Supplementary file 1 [file behavsci-14-01144-s001.zip › Figure S1.png]
